# Morphological awareness and its role in early word reading in English monolinguals, Spanish–English, and Chinese–English simultaneous bilinguals

**DOI:** 10.1017/s1366728922000517

**Published:** 2022-08-18

**Authors:** Rebecca A. Marks, Danielle Labotka, Xin Sun, Nia Nickerson, Kehui Zhang, Rachel L. Eggleston, Chi-Lin Yu, Yuuko Uchikoshi, Fumiko Hoeft, Ioulia Kovelman

**Affiliations:** 1 Department of Psychology, University of Michigan, Ann Arbor, MI 48109, USA; 2 Department of Brain and Cognitive Sciences, Massachusetts Institute of Technology, Cambridge, MA 02139, USA; 3 MGH Institute of Health Professions, Boston, MA 02129, USA; 4 School of Education, University of California Davis, Davis, CA 95616, USA; 5 Department of Psychiatry and Weill Institute for Neurosciences, University of California San Francisco, San Francisco, CA 94143, USA; 6 Department of Psychological Sciences and Brain Imaging Research Center (BIRC), University of Connecticut, Storrs, CT 06269, USA; 7 Haskins Laboratories, New Haven, CT 06511, USA

**Keywords:** Morphological awareness, word reading, literacy development, bilingualism, bilingual transfer

## Abstract

Words’ morphemic structure and their orthographic representations vary across languages. How do bilingual experiences with structurally distinct languages influence children’s morphological processes for word reading? Focusing on English literacy in monolinguals and bilinguals (*N* = 350, ages 5–9), we first revealed unique contributions of derivational ( *friend-li-est*) and compound (*girl-friend*) morphology to early word reading. We then examined mechanisms of bilingual transfer in matched samples of Spanish–English and Chinese–English dual first language learners. Results revealed a principled cross-linguistic interaction between language group (Spanish vs. Chinese bilinguals) and type of morphological awareness. Specifically, bilinguals’ proficiency with the type of morphology that was less characteristic of their home language explained greater variance in their English literacy. These findings showcase the powerful effects of bilingualism on word reading processes in children who have similar reading proficiency but different language experiences, thereby advancing theoretical perspectives on literacy across diverse learners.

## 1. Introduction

How does bilingual language experience influence the word reading process? Dual first language learners are exposed to their two languages simultaneously in early childhood, within the sensitive period of brain development for language function (Hager & Müller, 2015; Weber-Fox & Neville, 1996). At the same time, two languages housed within a single cognitive system are known to interact (Chung, Chen & Geva, 2019; Petitto, 2009). Here, we investigate the nature of this cross-linguistic interaction and its effect on word reading development through the lens of morphology. Although all words are made of morphemes, the smallest units of meaning, there is substantial cross-linguistic variability in words’ morphological structure and how it maps onto print. To uncover the mechanisms underlying bilingual transfer, we focus on children’s lexical morphological awareness – the branch of morphology concerned with forming new words – and its role in English literacy among young Spanish–English and Chinese–English simultaneous bilinguals. In the present study, we ask: how does bilingual experience with distinct morphological structures, such as derivational morphology in Spanish, or lexical compounding in Chinese, influence the role of morphological awareness in English reading? We first address lingering questions around the role of morphology in early English word reading (Study 1) before turning to our core inquiry into bilingual transfer of morphology in the context of word reading development (Study 2).

### 1.1 Theories of bilingual transfer and reading development

A bilingual’s two languages interact in the mind and brain, influencing literacy development. Theories of bilingual transfer such as the Interactive Transfer Framework (Chung et al., 2019) posit that a bilingual’s two languages are developmentally interconnected, and that the nature of cross-language interactions vary as a function of cognitive, linguistic, and environmental factors. The Interdependence Continuum framework (Proctor, August, Snow, Barr & Proctor, 2010) further suggests that cross-linguistic transfer is most likely at points of similarity between a bilingual’s two languages. Some skills, such as phonological awareness, are thought to be relatively language general, and thus likely to transfer across languages (Marks, Satterfield & Kovelman, 2022). Conversely, a child’s knowledge of morphology – or the underlying structure of words – depends on their knowledge of the lexical units in a given language, and the rules that govern how these units can be combined (Zhang & Ke, 2020). Importantly, and as we will discuss in detail in section 1.2, there is great variability across languages in words’ morphemic structure and how it is reflected in print. Morphological transfer depends on a bilingual’s language-specific knowledge as well as the similarity in linguistic structure of their two languages. Morphology therefore provides a unique opportunity to examine the linguistic processes underlying bilingual transfer between structurally distinct languages.

Much of our knowledge of the effects of bilingualism on English literacy has been informed by studies of English language learners (ELLs), or children who have high proficiency in a heritage language and are beginning to acquire English through formal schooling. Nevertheless, transfer may vary as a function of a learner’s proficiency (Chung et al., 2019), the linguistic structure of the specific language pairings, and points of similarity between the two (Proctor et al., 2010). This leaves a gap in our understanding of bilingual transfer among children who vary in points of structural similarity between their two languages and have a relatively high proficiency in those languages.

Dual first language learners, those exposed to two languages within the early sensitive periods of brain development for language function, are generally considered to have been afforded the opportunity to build maximally language-specific (or ‘monolingual-like’) linguistic and orthographic representations for each of their languages (Marks et al., 2022). At the same time, a growing body of research predicts cross-linguistic interactions that make even the earliest bilingual learners distinct from their monolingual peers (Ip, Hsu, Arredondo, Tardif & Kovelman, 2016). Here we explore the cognitive basis of cross-linguistic interactions in a sample of dual first language learners, specifically within the context of learning to read.

### 1.2 The role of morphology in reading across languages

Morphological awareness plays a role in learning to read across languages and orthographies (Kuo & Anderson, 2006). The Reading Systems Framework (Perfetti & Stafura, 2014) situates morphology as part of the lexical – as each word is made up of one or more morphemes, morphology should play an important role in single word identification. However, as the process of learning to read varies across languages and learning contexts, the precise contributions of morphological awareness to reading development may vary. In particular, Psycholinguistic Grain Size Theory (Ziegler & Goswami, 2005) suggests that orthographies differ in the size of the linguistic unit that is key for reading success. This variation is due, in part, to cross-linguistic differences in how units of sound and meaning map onto letters, as well as the underlying morphological structure of a given language.

In the case of Spanish, individual sounds map consistently onto units of print. Because of this phonological transparency, words could hypothetically be decoded at the level of individual sound-to-letter mappings. However, this process would be effortful and taxing, as Spanish roots are often polysyllabic, and words are frequently polymorphemic. Spanish is rich with derivational morphology and words often include multiple affixes, such as in the word *person*+*al*+*idad,* meaning *personality*. Many Latin cognates are shared between English and Spanish, including both the derivational principles and the affixes themselves, as well as entire word forms (e.g., *communication/comunicación*). Spanish also makes use of lexical compounding to create words, although compounding is relatively less productive and more analytically complex than in English, involving morphological adjustments to both roots. For instance, *rompecabezas* (puzzle) is composed of *rompe,* a third person singular present of *romper* (to break), and *cabezas,* a plural of *cabeza* (head).

Compared to young English readers, young Spanish readers are thus tasked with recognizing complex, polysyllabic words, often with multiple morphemes. The transparent sound-to-letter mapping of Spanish may allow children to use smaller grain sizes (individual phonemes) to access composite morphemes early in reading acquisition (e.g., Manolitsis, Grigorakis & Georgiou, 2017), which then aids children’s recognition of long, polysyllabic words. Accessing larger morphemic units in print may even serve as a mechanism for children with reading difficulties or phonological impairments (Portuguese: Oliveira, Levesque, Deacon & da Mota, 2020; Italian: Suárez-Coalla & Cuetos, 2013).

In contrast, the written characters in Chinese correspond to units of meaning (morphemes), rather than units of sound. Furthermore, over 90% of Chinese words are lexical compounds, as in *snow*+*man* (雪人, or literally *snow person*). Logically, lexical knowledge, such as morphological awareness or vocabulary, are critical predictors of Chinese literacy. Although morphology contributes to reading across languages, the transparent morpheme-to-print mapping of Chinese makes morphological awareness particularly important. For instance, a cross-cultural study of 2^nd^ grade readers of English, Korean, and Chinese revealed different associations between phonological, morphological, and vocabulary knowledge, and their contributions to word recognition across languages (McBride-Chang et al., 2005). In English, phonology was most closely correlated with word reading, whereas in Chinese, morphology and vocabulary were more strongly correlated with word reading than phonological awareness. Additionally, greater awareness of compound morphology in kindergarten is associated with better single word/character reading, and steeper growth trajectories in reading over time (Lin, Sun & McBride, 2019).

English uses both derivational morphology and lexical compounding to form new words. This creates a point of contact at which morphological awareness could potentially transfer for both Spanish–English, and Chinese–English bilinguals. It is therefore possible that children’s bilingual experiences may affect their sensitivity to specific morphemic structures, influencing the reading process in English (Pasquarella, Chen, Lam, Luo & Ramírez, 2011; Ramírez, Chen, Geva & Luo, 2011). However, the role of morphology in early English reading remains somewhat less clear, due in part to the notoriously inconsistent sound-to-letter mappings. We must therefore address lingering questions around the role of morphology in early English literacy.

English word reading presents somewhat of a paradox, due to the inconsistency of sound-to-letter mapping. This is because English orthography is morpho-phonological; spellings in English are based on both units of sound and units of meaning (Carlisle & Addison, 2005). In some cases, one phoneme might be spelled multiple ways (such as the */k/* sound in *castle, kitten, locker,* and *echo*). In other cases, spelling might remain consistent across words to maintain the underlying morphemic structure, even when the phonology changes (e.g., *music-musician* or *heal*-*healthy*). As a result, Psycholinguistic Grain Size Theory suggests that English readers may need to rely on larger linguistic units, such as morpho-syllables, to successfully read words (Ziegler & Goswami, 2006). Yet at the same time, these larger grains may be difficult to access, given the phonological and orthographic complexity of the language.

Theoretical models suggest that young English readers progress through several overlapping developmental phases as they learn to map orthography to word sounds (Ehri, 1995, 2014). As children acquire alphabetic knowledge, they begin to learn the connections between sounds and letters. Once they have mastered sound-to-print correspondences and can reliably decode unfamiliar words by mapping individual sounds to letters, they begin to consolidate groups of letters into larger syllabic or morphemic units. In Ehri’s model of sight word reading development (2005), English readers begin to recognize morphemic units in the final phase of development. Some studies have suggested that morphological awareness begins to contribute to word recognition only once children are proficient word readers and begin the transition from heavy reliance on sound-to-letter decoding towards more efficient strategies of rapid morpho-syllabic recognition (e.g., Singson, Mahony & Mann, 2000). Yet, given that every word consists of one or more morphemes, we might expect morphology to play a role in beginning, as well as more proficient, English word reading.

### 1.3 Morphological awareness and emerging English literacy

Children’s understanding of morphology in English begins to emerge in infancy, and continues to mature through middle and high school (Kuo & Anderson, 2006). First, children begin to recognize and master the rules governing inflectional morphology, which uses a limited number of morphemes to indicate grammatical function (e.g., *create-s, creat-ing, creat-ed*). Young children also learn to manipulate morphemes in order to create new words, either through lexical compounding or derivation. In English, compounding emerges first. Children as young as 18 months create novel lexical compounds by combining two words to fill gaps in their vocabulary (Clark, 1993), such as *nose-bangs* for a moustache. An understanding of derivational morphology (e.g., *re-create, creat-ive*) emerges slightly later and has a longer developmental trajectory (Kuo & Anderson, 2006).

Numerous studies have suggested that morphological awareness makes an increasing contribution to word reading throughout elementary and middle school, particularly among students in 3^rd^ grade and above (e.g., Carlisle & Kearns, 2017; Roman, Kirby, Parrila, Wade-Woolley & Deacon, 2009; Singson et al., 2000). In contrast to more advanced readers, it has been relatively difficult to characterize the relation between morphological awareness and concurrent word reading in younger children. Many scholars have combined inflectional (grammatical) and derivational morphology in their tasks for young children, revealing promising but inconsistent relationships between morphological awareness and early English reading in kindergarten through 2^nd^ grade across a variety of methodological approaches (Apel, Diehm & Apel, 2013; Kirby et al., 2012; Law & Ghesquière, 2017; Wolter, Wood & D’zatko, 2009). Notably, the role of compound morphology remains largely unexplored. Lexical compounding is one of the earliest emerging morphological skills (Clark, 1993), yet tests of compounding are missing from the lions’ share of English morphological awareness tasks. We therefore begin by clarifying the role of both derivational and compound morphological awareness in early English literacy during this uncertain developmental period, before turning to questions of bilingual transfer.

### 1.4 The present study

The present study asks: how does bilingual experience with distinct morphological structures, such as derivational morphology in Spanish, or lexical compounding in Chinese, influence the role of morphological awareness in English reading? To answer this bilingual question, we first addressed the lingering question on the relation between morphological awareness and word reading in English. In particular, we investigated children’s awareness of lexical morphology, or how morphemes can be combined to create words (e.g., *light+ly, high+light*), rather than their sensitivity to grammatical inflections (e.g., *light+s*). We thus developed and validated a measure tapping into children’s awareness of derivational and compound morphology with a large, linguistically diverse sample of children, ages 5–9. In Study 1, we tested the prediction that derivational and compound morphological awareness would be related to children’s concurrent word reading skills in kindergarten through 3^rd^ grade. In Study 2, we examined potential bilingual differences in the contribution of morphological awareness to English word reading between Spanish–English bilingual, Chinese–English bilingual, and monolingual English groups. We hypothesized that dual-language proficiency in Spanish or Chinese would have a contrasting, language-specific impact on children’s general linguistic systems, thereby influencing the reading processes in English. In particular, we predicted that experience with Spanish derivational morphology would transfer directly to support English derivation, which is governed by similar principles and shared structures, while experience with Chinese compound morphology would transfer to English compound awareness; these points of contact between English and bilinguals’ heritage language would influence their English morphological awareness, impacting English word reading mechanisms. Together, Study 1 and Study 2 shed light on the contribution of morphological awareness to early reading across linguistically diverse learners, and reveal language-specific transfer effects in Spanish–English and Chinese–English bilinguals.

## 2. Method

### 2.1 Participants and procedure

Participants were recruited as part of two larger neuroimaging studies of bilingual reading development from a college town in the Midwestern United States and a large urban center on the West coast. We intentionally recruited monolingual and bilingual populations, targeting heritage language schools and bilingual community centers. According to parental reports, half of our sample spoke a language other than English at home (22% Spanish, 25% Chinese, 3% other). All children were also proficient English speakers with age-appropriate vocabulary knowledge, as defined by standard scores above 80 on the Peabody Picture Vocabulary Test (D. M. Dunn, 2018; L. M. Dunn & Dunn, 2007).

We collected data from a large, socio-linguistically diverse sample of *N* = 396 children, ages 5–9. All participating children were in grade 3 or below. To be included in data analysis, children were required to be proficient English speakers with at least elementary word reading ability (details below). This left a final sample of *N* = 350 children (190 boys, 160 girls; *M*_age_ = 7.40 years old, *SD* = 1.06). Participants were of varied racial and ethnic backgrounds: the sample was 35% White, 27% Asian, 20% Latinx, and 2% Black. 16% of children came from multiracial or multiethnic backgrounds. Children came from highly educated homes, with nearly 88% of mothers having a college degree or above. Demographic characteristics for all participants are presented in the Supplement.

This full sample of *N* = 350 children informed our research questions about English morphology development and English literacy more broadly (Study 1). To answer our specific questions about bilingual transfer from contrasting languages, we limited our inquiry to children who were dual first-language speakers of English, and either Spanish or Chinese (Study 2). We used the MatchIt package in R to identify subsamples of English monolinguals, Spanish–English bilinguals, and Chinese–English bilinguals with equivalent English vocabulary knowledge and word reading skills (Table 3). This resulted in three matched groups of *N* = 69 each, leading to a sample of *N* = 207. More details about bilinguals’ language exposure, and dual language proficiency will follow in Results 3.2.

### 2.2 Assessing derivational and compound morphological awareness

To answer our question about bilingual transfer, we developed a morphology measure that targeted children’s sensitivity to derived and compound morphemic structures. One well-established task relies on the decomposition model (Carlisle, 2000), in which children are asked to extract the base of a multimorphemic word to complete a sentence. For the present study, we developed a measure based on the Extract the Base task by Goodwin and colleagues (Goodwin, Huggins, Carlo, Malabonga, Kenyon, Louguit & August, 2012), which was extensively piloted and validated with a large group of linguistically diverse 3^rd^ to 5^th^ graders.

We modified this measure to ensure that it was well-suited to this research, first by making it more accessible to young children, and then by adding items that tapped into awareness of compound morphology. To ensure that our measure was accessible to pre-readers as well as readers, the task was administered orally, with no visual or written component. The experimenter told each participant, “I will say a word, and then you will use *part* of that word to help me finish my sentence.” The experimenter then said a multimorphemic word, followed by an incomplete sentence (e.g., *Friendly. She is my best*..._____). Children were expected to complete the sentence using the root word (e.g., *friend*). Participants received feedback on this training item. No feedback was given on subsequent testing items, which were presented in a fixed order with increasing levels of difficulty. Testing was discontinued if the child made 10 consecutive errors.

We made a few notable changes to the specific items presented in the original Extract the Base task (Goodwin et al., 2012). First, we expanded the assessment to include compound morphology in addition to derivational morphology. We further redesigned existing task items to place all target words at the end of a simple sentence, thereby reducing working memory load, and replaced later-acquired, academic vocabulary with earlier-acquired words. For instance, instead of asking children to extract the base *fear* from *fearful,* or *dense* from *density,* children extracted *color* from *colorful* and *person* from *personality.* This change was intended to make the task more accessible to young children.

Our final Early Lexical Morphology Measure (ELMM) consisted of 40 items (15 compound, 25 derived). Five derivational items were identical to those in Goodwin et al.’s (2012) measure. Six were modeled on items from Goodwin and colleagues (2012) but used a modified sentence prompt that was more accessible to young children. For instance, instead of the prompt, “*Combination.* Which chemicals should I ___?” our participants heard, “Which colors should the painter ___ [*combine*]?” The final task also included 29 newly developed items, 15 of which assessed compound morphology (see Appendix). Fifteen items overall required a phonological shift (e.g., *discussion* – *discuss*).

The 25 derived items had a higher average age of acquisition than the 15 compound items and captured a broader range of morphological competence, reflecting the higher productivity of derivation in English more broadly. However, because of our focus on the role of compound and derivational awareness in young bilingual readers, the ELMM also contains a matched subset of 13 compound and 13 derived items with base words acquired prior to age 6. An example of an early-acquired compound item is, “*Sidewalk.* The baby is learning how to ___ [*walk*]” while an early acquired derived item is, “*Noisy*. Did you hear that ___ [*noise*]?” The multimorphemic prompt words in this subset had a mean age of acquisition (Kuperman, Stadthagen-Gonzalez & Brysbaert, 2012) of 5.60 (*SD* = 1.50), while the base words had a mean age of acquisition of 4.75 (*SD* = 1.25). Independent sample *t*-tests confirmed that there were no significant differences between root morphemes in derived versus compound items in terms of their age of acquisition (*t*(24) = 1.62, *p* = .119; Kuperman et al., 2012), frequency in child-directed speech (*t*(24) = −1.43, *p* = .165; MacWhinney, 2000), nor frequency in adult speech (*t*(24) = −1.07, *p* = .297; Davies, 2008). Although most of our analyses use participants’ performance on the full 40 item ELMM, we present data from these 26 matched, early acquired derivations and compounds when testing *a priori* hypotheses about bilingual transfer of derivational versus compound morphology. This methodological choice allows us to better distinguish children’s awareness of composite morphemes and the rules governing word construction from their vocabulary knowledge.

We first validated the ELMM with a large, socio-linguistically diverse sample of 340 monolingual, bilingual, and multilingual speakers (see Results 3.1). We then compared morphological awareness, as well as the role of MA in English reading, in three matched groups of English monolinguals, Chinese–English bilinguals, and Spanish–English bilinguals, using standardized measures of English language and literacy.

### 2.3 Standardized measures of language and literacy

#### Receptive vocabulary

Receptive vocabulary was assessed using the Peabody Picture Vocabulary Test (PPVT). Participants on the West coast were assessed using the PPVT-4 (Dunn & Dunn, 2007) as part of a larger, longitudinal study that began in 2015. Data collection at the Midwestern site began in 2019, using the updated PPVT-5 (Dunn, 2018). Of the *N* = 396 children tested, 17 bilingual and one monolingual participant were excluded due to low English vocabulary (standard score below 80). This exclusion criterion allowed us to focus our analyses on the literacy mechanisms of readers who had achieved grade-appropriate English proficiency.

#### Single word reading

Single word reading was assessed using the Letter-Word Identification subtest from the Woodcock Johnson IV Tests of Achievement (Schrank, Mather & McGrew, 2014). The first test items require children to identify letters, and later items ask children to read single words of increasing complexity. Approximately 70% of the test items are monomorphemic words, while 30% are multimorphemic. In order to be eligible for the present study, children were required to have a raw score of 14 or above, indicating that they could successfully name letters and identify at least four high frequency words such as *dog* or *the*. Twenty-eight children (15 monolinguals, 13 bilinguals) were excluded due to low word reading ability, leaving a final sample of *N* = 350.

#### Reading comprehension

Reading comprehension was assessed using the Woodcock-Johnson Passage Comprehension subtest (Schrank et al., 2014). This task measured comprehension of connected text. For beginning readers, the Passage Comprehension task is heavily supplemented by pictures, while more advanced items require children to read a sentence or passage and fill in a missing word. As prior research has suggested that word reading and reading comprehension may be best understood as a single construct prior to grade 3 (Lonigan & Burgess, 2017), the present study only examines word reading as our dependent variable of interest. Nevertheless, we present correlations with reading comprehension as well, given the well-documented association between morphological awareness and reading comprehension in more advanced readers.

#### Phonological awareness

Phonological awareness was assessed using the Elision subtest of the Comprehensive Test of Phonological Processing (CTOPP-2; Wagner, Torgesen, Rashotte & Pearson, 2013). Children are asked to pronounce a word while removing a phonetic unit. Among the first nine items, seven asked children to extract a morpho-syllable from a compound word (e.g., “Say *toothbrush* without saying *tooth*”), and two asked children to extract a syllable that was not meaningful (e.g., “Say *spider* without saying *spy*”). Items then progressed to the level of single phonemes (e.g., “Say *winter* without saying */t/*”). Scaled scores on this phonology measure have a mean of 10; scaled scores between 8–12 fall within the typical developmental range.

#### Heritage language assessments

We also conducted an extensive battery of heritage language assessments including phonological, morphological, and word reading skills. For the present study, which targets group differences in English word reading specifically, we present only children’s Spanish or Chinese vocabulary and morphological awareness. Please see Sun, Zhang, Marks, Nickerson, Eggleston, Yu, Chou, Tardif, and Kovelman, I. (2021) as well as the Supplementary Materials for extensive details about the bilingual language assessments, participants’ literacy skill in each language, and evidence of direct transfer from children’s heritage language to English.

Heritage language vocabulary was assessed using the Spanish and Chinese versions of the PPVT (Dunn, Padilla, Lugo & Dunn, 1986; Lu & Liu, 1998). Importantly, these assessments are both standardized, but the standard scores are normed based on different populations. As part of a larger project, some participants in the current study completed an additional morphological awareness task in Spanish or Chinese. This was an auditory oddball task, in which children heard three words: two that shared a morpheme (e.g., classroom and bedroom), and one with a phonological distractor (e.g., mushroom). In Spanish, an example triplet includes the words *automóvil* (automobile), *autopartes* (car parts), and the distractor *autoridad* (authority). In Chinese, an example triplet includes the words 眼镜 (*yan3 jing4*, eyeglasses), 墨镜 (*mo4 jing4*, sunglasses), and the distractor 安静 (*an1 jing4*, quiet). These example items all demonstrate triplets in which two words share a root morpheme; however, the tasks also contain items that share a derived affix. Children’s heritage language morphological awareness is presented in terms of the percentage of correct items out of 32 experimental trials.

## 3. Results

### 3.1 Study 1: associations between morphology and word reading

#### Descriptive analyses

All 350 eligible children, ages 5–9, participated in Study 1. Twenty percent of this sample was enrolled in junior kindergarten or kindergarten (*N* = 70, *M*_age_ = 6.03), 39% was in 1^st^ grade (*N* = 137, *M*_age_ = 7.05), 27% was in 2^nd^ grade (*N* = 93, *M*_age_ = 8.17), and 14% was in 3^rd^ grade (*N* = 50, *M*_age_ = 8.92). The sample included monolingual English speakers and dual first-language learners who spoke a language other than English at home. The break-down of children’s home language background by grade is provided in Supplemental Table 2.

Participants had high-average English language and literacy skills, with mean standard scores ranging from 104 to 112. Table 1 provides descriptive statistics as well as the Pearson correlations between each measure. Both derivational and compound awareness were significantly associated with literacy outcomes, with correlation coefficients ranging from .52 - .71 (Table 1). The strongest relationship was between derivational morphological awareness and reading comprehension, *r*(326) = .71, *p* < .001. Fisher *r*-to-*z* transformations revealed no meaningful difference in the strength of association between derivations and compounds to word reading (*z* = 1.48, *p* = .069); however, derivational awareness was more strongly associated with reading comprehension (*z* = 1.76, *p* = .040).

#### Validity of the early lexical morphology measure

To examine the dimensionality and internal consistency of the ELMM, we ran two confirmatory factor analyses in lavaan v0.6–9 (Rosseel, 2012) using a weighted least square mean and variance adjusted (WLSMV) estimator. The goal of these analyses was to compare a two-factor model, in which derivations and compound items loaded onto separate constructs, as opposed to a one-factor model with a single underlying morphological awareness construct. We entered binary data for each of the 40 ELMM items: participants received a 1 for a correct response, and a 0 for an incorrect response or after testing had been discontinued. The two-factor model yielded an excellent fit for our data (χ^2^ (739, *N* = 346) = 671.79, *p* = .963, RMSEA estimate = .03, CFI = .94, TLI = .94, SRMR = .05), and had a significantly better fit than the one-factor model, $\chi_{diff}^{2}\left(1\right)=11.83$, *p* < .001. This suggests that derivational and compound morphological awareness may be best understood as two related but distinct cognitive abilities. The Cronbach’s alpha reliability coefficient was .93, indicating good internal consistency.

#### Morphological awareness from kindergarten to 3rd grade

**Fig. 1.**
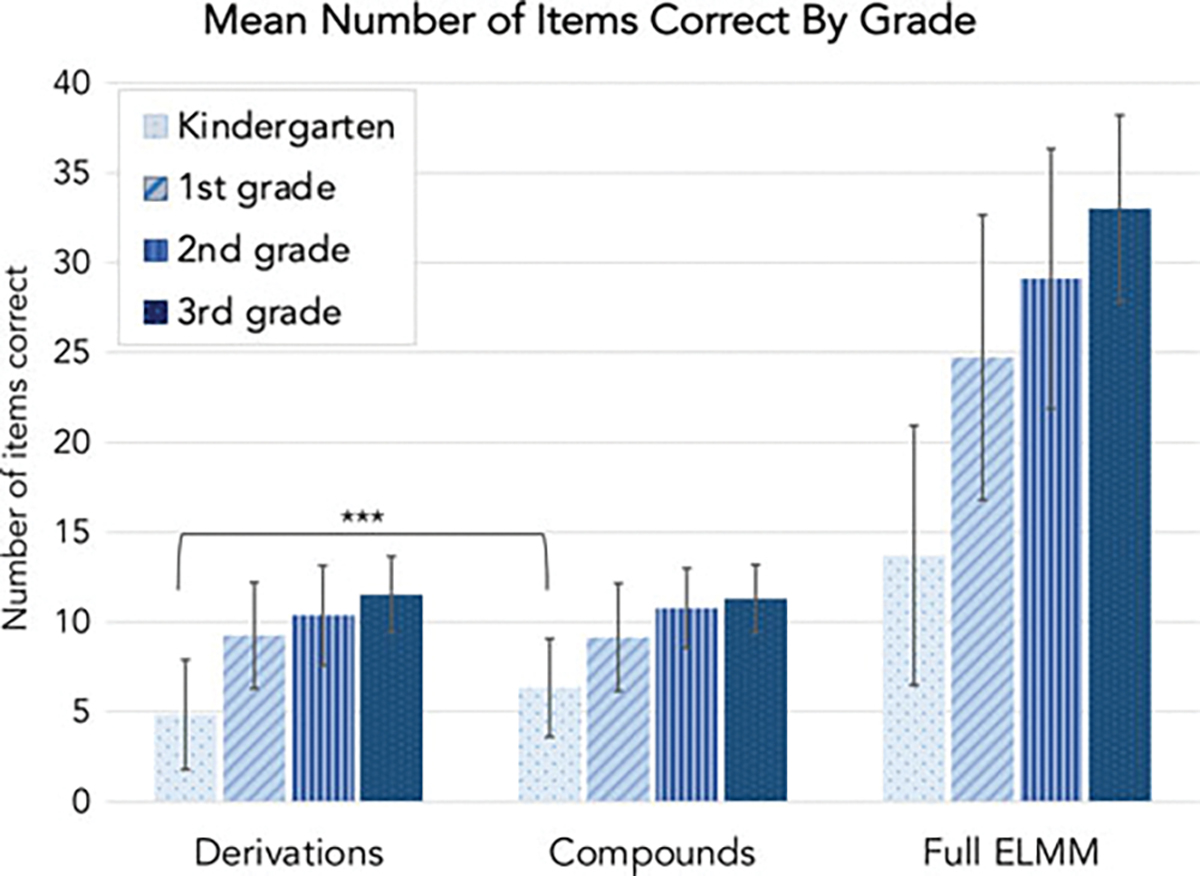
Bar graph depicting performance on morphological awareness task at each grade level. Derivations and Compounds: *N* = 13 items each with matched, early acquired morphemes; Full ELMM: *N* = 40 items. Vertical bars indicate standard deviations.

We found that the ELMM task was accessible to 5-year-old kindergarteners as intended, sensitive to developmental differences in children ages 5–9, and reliable across all grade levels (K: α = .89, 1^st^: α = .90, 2^nd^: α = .91, 3^rd^: α = .78). A one-way analysis of variance (ANOVA) confirmed significant differences in performance by grade (*F*(3, 344) = 82.25, *p* < .001). Planned *t*-tests revealed a significant difference in performance between junior kindergarteners or kindergarteners (*M* = 13.50, *SD* = 7.22) and 1^st^ graders (*M* = 24.72, *SD* = 7.93), corresponding to the onset of literacy instruction; *t*(205) = 9.93, *p* < .001, *d* = 1.49. After the start of schooling, children showed a steady developmental increase in performance (see Figure 1, Supplemental Table 3). Additional *t*-tests also revealed significant differences in children’s total raw score between 1^st^ and 2^nd^ grade (*t*(226) = 3.77, *p* = .001, *d* = 0.52), as well as between 2^nd^ and 3^rd^ (*t*(139) = 3.13, *p* = .002, *d* = 0.56). All *t*-tests survive Bonferroni correction for 3 comparisons (α = .017).

We also conducted paired *t*-tests to examine age-related changes in performance on derivational affixes as compared to compound morphology (Figure 1). For this analysis, we used 13 derived and 13 compound items, matched on age of acquisition (Kuperman et al., 2012) and frequency (Davies, 2008; MacWhinney, 2000). This choice allowed us to examine developmental differences in morphological competence with early-acquired roots and affixes. Because prior research suggests children’s awareness of English lexical compounding may emerge earlier than skill with derivations (Clark, 1993), we hypothesized that our younger participants would demonstrate higher accuracy on compound items. Indeed, children’s accuracy on compound items in pre-kindergarten or kindergarten (*M* = 6.34, *SD* = 2.73) was significantly better than their accuracy on derived items (*M* = 4.84, *SD* = 3.06; *t*(69) = 5.69, *p* < .001, *d* = 0.51). This significant difference in accuracy on compound vs. derivational items was not apparent in later grades (all *p*s > .05).

#### Associations between morphology and word reading

We hypothesized that compound and derivational morphological awareness would make independent contributions to children’s word reading ability. We therefore conducted a hierarchical regression to the relation between morphology and single word reading in the full sample of *N* = 350 participants (see Table 2). At step 1, we entered children’s age, maternal educational attainment, and bilingual status (0 = monolingual, 1 = bilingual) as control variables. At step 2, we entered vocabulary knowledge and phonological awareness, two well-established predictors of word reading. At step 3, we entered children’s derivational awareness scores out of 25, and their compound awareness scores out of 15. Twenty-two participants had missing data for at least one of the included variables, leaving a test sample of *N* = 328. Results showed that both derivational (*β* = .23, *t* = 3.61, *p* < .001) and compound (*β* = .11, *t* = 2.01, *p* = .045) morphological awareness were significant unique predictors, accounting for an additional 3.9% of the variance in word reading (*F*(2, 321) = 19.08, *p* < .001). After adding derivational and compound morphology to the model, vocabulary was no longer significant. In sum, the ELMM effectively captured developmental differences in morphological awareness in children ages 5–9, and revealed robust relationships between derivational morphology, compound morphology, and word reading from kindergarten through grade 3.

We then conducted a post-hoc analysis to examine the contributions of morphological awareness to literacy in kindergarteners and 1^st^ graders (*N* = 207), and in 2^nd^ and 3^rd^ graders (*N* = 143) separately. This analysis extends prior literature that has suggested that morphological awareness plays a lesser role in early reading development and makes an increasingly large contribution over the course of elementary and middle school (Roman et al., 2009; Singson et al., 2000). Hierarchical regressions revealed that morphology made a similar unique contribution to single word reading in the younger grades (Δ*R*^2^ = .045, *p* < .001) as in the older grades (Δ*R*^2^ = .041, *p* = .001). With reduced power in these two smaller samples, derivational awareness emerged as a significant unique predictor of word reading in both analyses (younger: *β* = .24, *t* = 2.72, *p* = .007; older: *β* = .21, *t* = 2.33, *p* = .021), while compound awareness did not. However, when derivational awareness was not included in the regression model, compound awareness made a similar unique contribution to word reading across groups (younger: *β* = .21, *t* = 3.49, *p* < .001; older: *β* = .19, *t* = 3.01, *p* = .003). Full regression tables for these analyses can be found in the Supplementary Materials (Tables S4–S7), demonstrating a similar association between morphological awareness in beginning as well as more advanced readers.

### 3.2 Study 2: Bilingual transfer effects on morphological awareness and English reading

The goal of Study 2 was to examine bilingual transfer effects on speakers of two structurally distinct languages. Drawing from the larger sample in Study 1, we limited our inquiry to children who were heritage speakers of Spanish or Chinese and had not been exposed to additional languages. Sixty-nine Spanish–English bilinguals and 80 Chinese–English bilinguals met these criteria. These bilingual participants all had at least one parent or primary caregiver who was a native speaker of either Spanish or Chinese. These caregivers had all been born outside of the United States, in a Spanish- or Chinese-speaking country (predominantly Mexico and mainland China). Child participants had been exposed to their heritage language since birth. Nearly 18% of these children attended language immersion public schools (8 Spanish–English immersion, and 16 Chinese–English immersion), while the remaining participants attended English-only general education programs. An additional 34% received some formal literacy instruction in their heritage language through extracurricular activities, such as a Saturday language school (18 Spanish, and 28 Chinese). An additional 27 Spanish-speaking parents and 2 Chinese-speaking parents reported that they were teaching their child to read at home in the absence of formal heritage language literacy instruction. All bilingual children were fluent in English, with mean vocabulary standard scores of 106.87 (*SD* = 14.69). Notably, bilinguals also had heritage language vocabulary within the typical range despite growing up in English-dominant communities in the United States (Spanish *M* = 108.60, *SD* = 16.20; Chinese *M* = 95.90, *SD* = 17.39). Bilinguals thus had relatively high and balanced dual-language proficiency.

We then identified a subsample of English monolinguals with similar English language and literacy skill to our bilingual participants, and no sustained exposure to other languages. To disentangle possible effects due to language background versus differences in English vocabulary knowledge or reading skill (Hammer et al., 2014), we used the MatchIt package in R (Ho, Imai, King & Stuart, 2011) to create three groups of English monolinguals, Spanish–English bilinguals, and Chinese–English bilinguals with matched English vocabulary and English word reading ability. The English monolingual and Chinese–English bilingual groups each had 69 participants, matched to the 69 eligible Spanish–English bilinguals, resulting in a total sample of *N* = 207.

Using these matched groups, we examined bilingual transfer in two ways. First, we compared differences in bilingual children’s awareness of English morphological structures that were shared across their two languages (i.e., derivations for Spanish–English bilinguals, and compounds for Chinese–English bilinguals) versus those that were dissimilar. Second, we examined how cross-linguistic experiences with typologically distinct morphologies might influence the relations between English morphological awareness and English literacy.

#### Descriptive analyses

Table 3 provides descriptive statistics of raw achievement scores across the three groups, and Table 4 provides the intercorrelations between language and literacy variables for each language group. To confirm that our three groups were well-matched, we conducted a one-way ANOVA which revealed no significant group differences in English vocabulary, phonological awareness, word reading, or morphological awareness. There were significant differences between the groups in age (*F*(2, 204) = 5.27, *p* = .006) and maternal education (*F*(2,195) = 6.02, *p* = .003). Nevertheless, these subsamples of English monolinguals, Spanish–English bilinguals, and Chinese–English bilinguals performed equivalently on all measures of raw English language and literacy skill. The two bilingual groups also performed within the typical range in Spanish and Chinese vocabulary; however, as these measures were normed on different populations, one cannot reliably compare vocabulary knowledge across languages.

As part of a larger study, a subsample of bilingual children in the matched groups also completed a heritage language task of morphological awareness. *N* = 52 Spanish bilinguals (*M* accuracy = 60.04%, *SD* = 16.62) and *N* = 43 Chinese bilinguals (*M* accuracy = 60.10%, *SD* = 14.69) completed this oddball task in their heritage language. There were no significant differences in accuracy between groups, *t*(93) = −0.02, *p* = .984. Although these data are not available for all Study 2 bilinguals, they suggest that the bilingual groups are likely well-matched in their heritage language proficiency as well as their English proficiency. Additional details about participants’ heritage language skill and the role of heritage language morphology in English word reading are available in Supplementary Materials.

Of note, however, are the different associations between language and literacy variables across language groups (Table 4). For instance, phonological awareness and word reading were more strongly correlated among Spanish–English bilinguals than the monolinguals (*z* = 2.15, *p* = .016), although there was no difference from the Chinese bilinguals (*z* = 1.10, *p* = .136). On the other hand, vocabulary and word reading were more weakly correlated among Spanish–English bilinguals than the other two groups (*z* = 1.65, *p* = .050). Also noteworthy are the correlations between the bilingual participants’ heritage language vocabulary and their English literacy skills. While Spanish vocabulary was significantly correlated with all English measures, Chinese vocabulary was only significantly associated with English compound awareness. The higher correlations between Spanish vocabulary and English measures likely reflects the closer relation between these two languages, and more frequent points of linguistic contact to facilitate transfer. In contrast, the correlations with Chinese vocabulary reflect a different transfer effect. Chinese vocabulary is more highly correlated with English skills associated with lexical or semantic knowledge (English vocabulary, reading comprehension, and compound morphological awareness). Word reading and derivational morphology were more weakly associated as these skills are not as easily transferable between English and Chinese. Nevertheless, heritage language morphological awareness was correlated with heritage language vocabulary as well as English morphological awareness and English literacy skill in both bilingual groups.

#### Performance on English derived vs. compound morphology

**Fig. 2.**
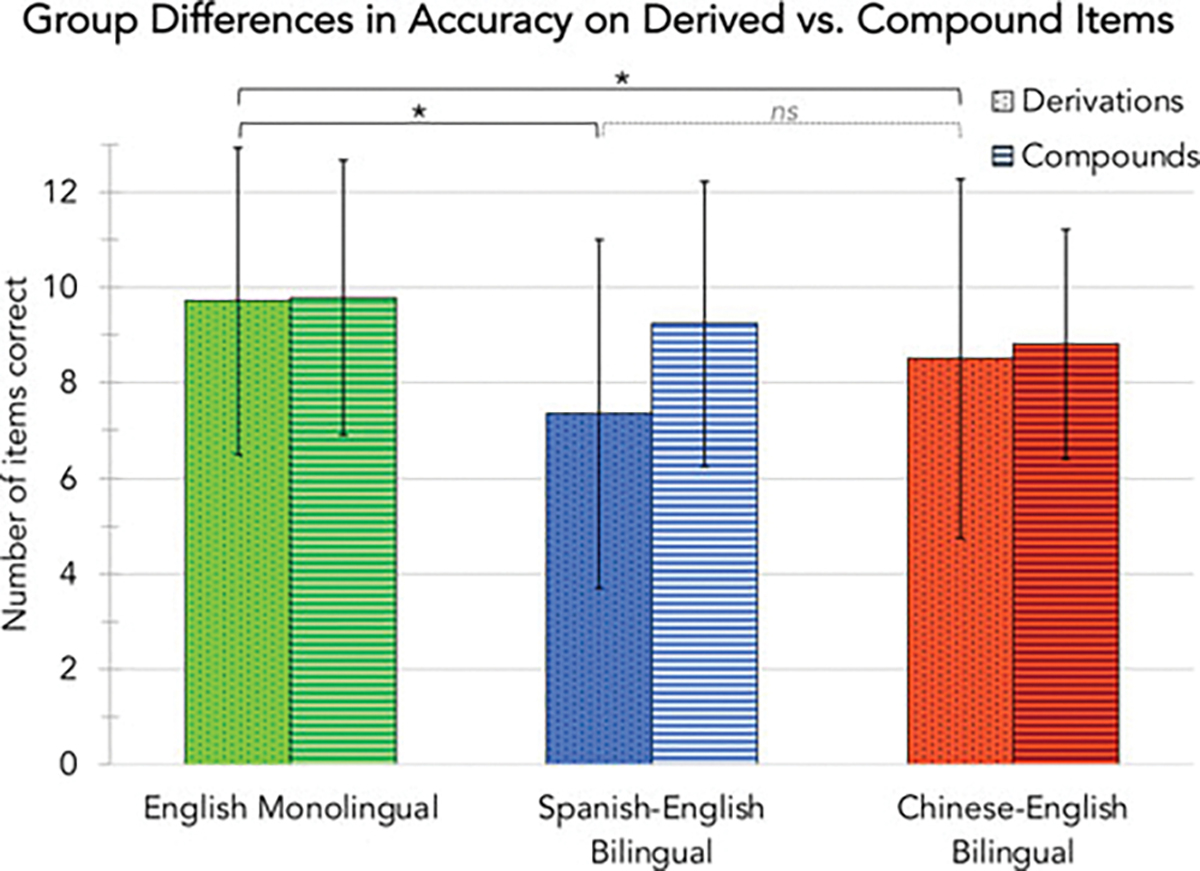
Bar graph comparing English monolinguals, Spanish–English bilinguals, and Chinese–English bilinguals on their accuracy with early-acquired derived and compound ELMM items. Language groups have matched English vocabulary knowledge and word reading ability. Vertical bars indicate standard deviations.

The first aim of Study 2 was to examine how children’s bilingual experiences with distinct morphological structures might influence their morphological awareness in English. Guided by theories of bilingual transfer, we compared Spanish–English and Chinese–English bilinguals’ performance on derivational vs. compound items of the ELMM. This analysis specifically considered the subset of 26 early acquired items, which included 13 derived and 13 compound items with similar frequency and age of acquisition. We hypothesized that bilingualism would alter a child’s general linguistic system, lexicon, and sensitivity to certain grain sizes, thereby influencing the reading process in English. Specifically, we predicted that Spanish–English bilingual children would show advantages in English derivational morphology, while Chinese–English bilingual children would show advantages in English compound morphology. However, independent sample *t*-tests revealed no differences between bilingual groups in terms of their accuracy on the matched subset of derived items *(M* Spanish = 8.35, *M* Chinese = 8.51, *t*(136) = −0.25, *p* = .801) or compound items (*M* Spanish = 9.25, *M* Chinese = 8.81, *t*(136) = 0.80, *p* = .425). Although both bilingual groups had significantly lower performance on derivational morphology than the monolingual group, they were not significantly different from one another (Figure 2). In other words, children’s awareness of compound and derivational morphology did not vary as a function of their specific bilingual experiences with derivationally-rich Spanish or compound-rich Chinese.

#### Associations between derivational awareness, compound awareness and English literacy

The second aim of Study 2 was to examine possible bilingual differences in the relation between English morphological awareness and English literacy. Might bilingual experience with structurally distinct languages influence the roles of derivational and compound morphological awareness in English reading? We hypothesized that compound and derivational morphological awareness would differentially contribute to children’s English reading as a function of their bilingual language backgrounds.

In parallel to Study 1, we conducted a multiple regression analysis to predict variance in English single word reading (Table 5) This model included language group (LG) as a factor with three levels (English monolingual, Spanish–English bilingual or Chinese–English bilingual), children’s ELMM score on derivations, and their score on compounds. Age, maternal education, and English vocabulary were included as covariates. All predictors were *z*-scored, and interaction terms were computed using the language group factor and the *z*-scored Derivations and Compounds variables.

Regression results are presented in Table 5, using both Spanish- and Chinese–English bilinguals as a reference group for clarity. We found that age, maternal education, and English vocabulary were all significant predictors of word reading. There was a significant effect of language group, in which both Spanish bilinguals (*b* = 0.25, *t* = 2.37, *p* = .019) and Chinese bilinguals (*b* = 0.38, *t* = 3.77, *p* < .001) differed significantly from the English monolinguals, but not from one another. Furthermore, findings revealed significant main effects of derivational awareness and compound awareness, as well as significant interactions between language group and derivational vs. compound morphology.

**Fig. 3.**
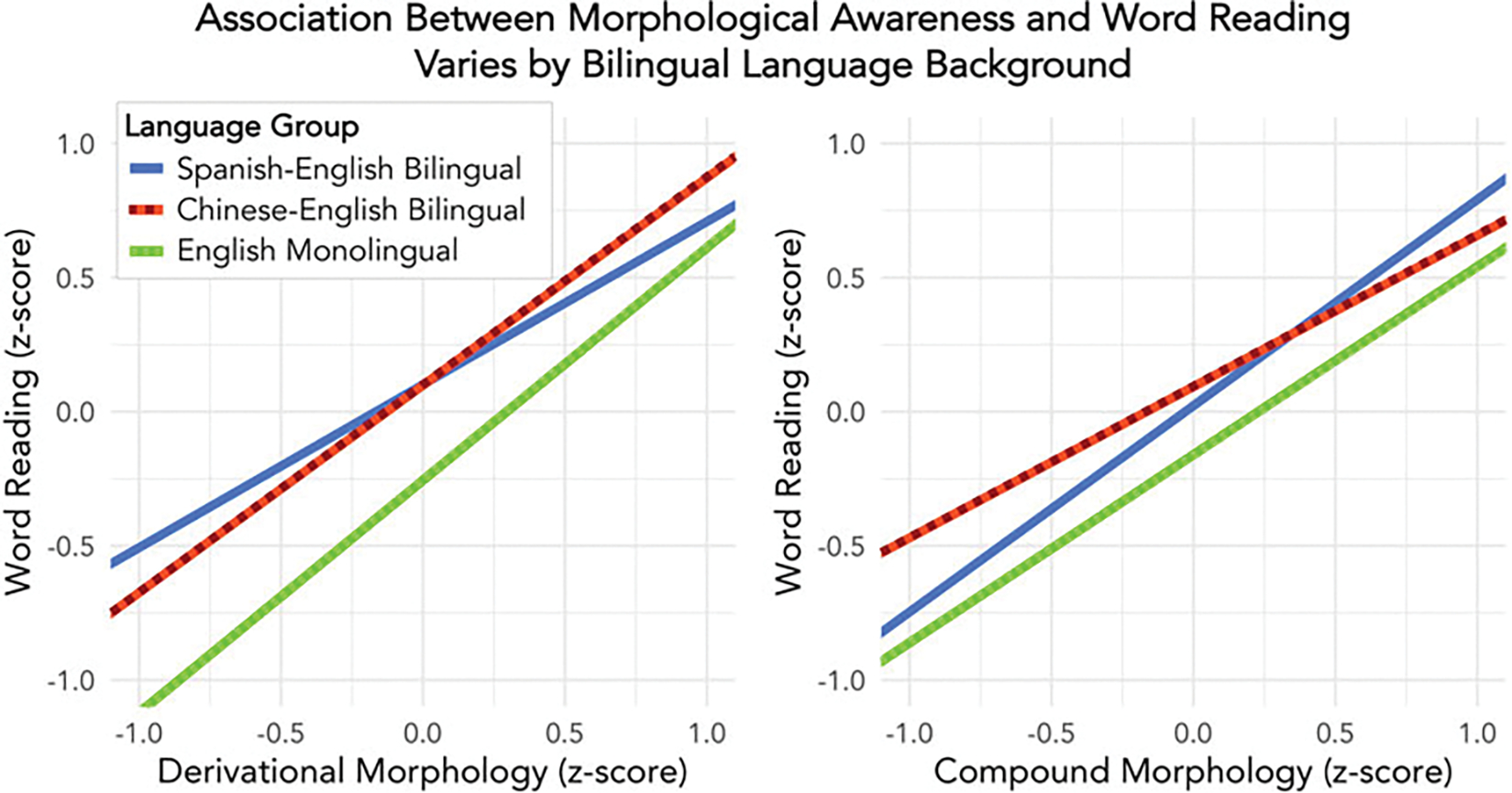
Line graph of interaction between language group (Spanish–English bilingual, Chinese–English bilingual, and English monolingual) and morphological awareness for each item type (derivational and compound morphology).

To decompose the significant interactions between language group and derivational vs. compound morphology (Figure 3), we examined the simple slopes of the morphological awareness variables across the bilingual groups. For Spanish–English bilinguals, compound awareness was significantly associated with English word reading (*b* = .48, *t* = 4.14, *p* < .001), while derivational morphological awareness was not (*b* = −.01, *t* = −0.10, *p* = .918). In contrast, for Chinese–English bilinguals, derivational morphological awareness was significantly associated with word reading (*b* = .37, *t* = 3.24, *p* = .001) while compound awareness was not (*b* = .07, *t* = 0.71, *p* = .481). In other words, only compound morphology explained unique variance in Spanish–English bilinguals’ word reading, while only derivational morphology explained unique variance in Chinese–English bilinguals’ word reading. The roles of derivational and compound morphology in monolingual English readers were similar to the Chinese–English bilinguals. English monolinguals had the steepest slope for derivational awareness (*b* = .54, *t* = 4.69, *p* < .001), although it was not significantly different from the Chinese–English bilinguals, while compound awareness was not significant (*b* = .10, *t* = 0.90, *p* = .369).

#### The role of heritage language morphological awareness in English word reading

Finally, we conducted a post hoc analysis to examine how heritage language morphological awareness might contribute to English word reading skill, after taking into account the contribution of English morphological awareness. We conducted two separate regression analyses, one for the Spanish–English bilinguals and one for the Chinese–English bilinguals who had completed the heritage language morphology task. Among Spanish–English bilinguals, English compound awareness and Spanish morphological awareness, but not English derivational awareness, were significantly associated with English word reading (Table 6). Among Chinese–English bilinguals, only English derivational awareness was significantly associated with English word reading (Table 7).

## 4. Discussion

The overarching goal of this inquiry was to examine the effects of early dual language experience on English morphological awareness and its relation to word reading in developing readers, ages 5–9. Using the novel Early Lexical Morphology Measure (ELMM), we observed a robust contribution of both derivational and compound morphological awareness to word reading across a large, linguistically diverse sample. Next, the findings revealed cross-linguistic influences of bilingualism on the relationship between children’s morphological awareness and learning to read: bilinguals’ proficiency with the type of morphology that was LESS characteristic of their home language explained greater variance in their English literacy.

The present findings showcase the effects of dual language experience on literacy development, even in populations of bilinguals who have relatively high levels of proficiency in both of their languages. On the surface, the two groups have similar levels of English reading skill. Yet our findings reveal underlying mechanistic differences in children’s word processing related to their heritage language backgrounds. These findings advance theoretical perspectives on literacy in monolingual and bilingual learners by clarifying the association between morphological awareness and early English literacy skill, as well as the cross-linguistic bilingual effects on this association.

### 4.1 Assessing derived and compound morphological awareness

Leveraging the body of knowledge on morphology development, we modified an existing measure to be maximally sensitive to children’s emerging derivational and compound morphological awareness between kindergarten and 3^rd^ grade. Our Early Lexical Morphology Measure was built upon the well-established Decomposition (Carlisle, 2000) or Extract the Base (Goodwin et al., 2012) task model (e.g., *Playful.* Let’s go outside and ___ [*play*]). To ensure accessibility for our youngest participants, we modified morphemic, sentential, and lexical features of the task. Most notably, we included lexical compounding, an early-emerging component of English morphological awareness. We additionally modified derivational items from the Extract the Base measure (Goodwin et al., 2012) so that all items were based on child-friendly root words (e.g., *noise, color* as opposed to *reduce, proceed*), and embedded these words at the end of short sentences to reduce working memory load. ELMM performance meaningfully captured variability in morphological awareness across a wide age range: there was no floor effect in 5-year-old kindergarteners, and no ceiling effect in 9-year-old 3^rd^ graders. To our knowledge, this is the first measure of lexical morphology appropriate across this age range. This represents an important methodological advancement in the field, as it captures the critical transition from “learning to read” to “reading to learn” in elementary school.

ELMM not only captured a steady increase in morphological awareness from kindergarten through 3^rd^ grade; it also revealed developmental differences in children’s faculty with compound versus derivational morphology more specifically. In kindergarten or pre-kindergarten, children were significantly better at extracting root morphemes from compound words (e.g., *rain* from *rainbow*), than from derived words (e.g., *quick* from *quickly*). This finding is closely aligned with recent work suggesting that young German readers are sensitive to compound lexical structure earlier than derivational prefixes or suffixes (Hasenäcker, Schröter & Schroeder, 2017). By 1^st^ grade, children in our study were able to extract root morphemes from derived and compound words equally well, and with evidence of further improvement in 2^nd^ and 3^rd^ grade. This dramatic change in derivational morphological awareness between kindergarten and 1^st^ grade may be related to the documented association between derivational vocabulary knowledge and schooling experience (Anglin, 1993). This finding enhances our understanding of the developmental trajectory of morphological knowledge or awareness, and reinforces the value of assessing compound morphology at the onset of schooling. By using early-acquired root words, a broad range of morphological constructions, and compound morphology in addition to derivations, we gain a clearer picture of children’s morphological awareness and the nature of its contribution to early reading development.

### 4.2 Clarifying the role of morphology in early English reading

Although prior work has revealed an increasingly robust association between morphological awareness and word reading throughout elementary and middle school (Carlisle & Kearns, 2017; Roman et al., 2009), the role of morphology in early English word reading has not been entirely clear. Among younger children, in kindergarten through 2^nd^ grade, associations between morphological awareness and early English word reading have been relatively inconsistent (Apel et al., 2013; Kruk & Bergman, 2013; Law & Ghesquière, 2017; Wolter et al., 2009). For instance, using different tasks of morphological awareness, both Apel and colleagues (2013) and Law and Ghesquière (2017) found that morphology was associated with concurrent word reading in kindergarten and 2^nd^ grade, but not 1^st^ grade.

The present study clarifies the associations between morphology and concurrent English literacy during this less certain developmental period prior to grade 3. Both compound and derivational morphological awareness significantly predicted children’s word reading ability, above and beyond phonological awareness, vocabulary, and demographic variables. When included simultaneously in regression models, derivational awareness explained substantially more variance in word reading, whereas compound awareness made a smaller contribution.

Importantly, morphological awareness contributed to word reading across grades and levels of reading ability. When we split our sample by grade, the ELMM task explained an additional 4.5% unique variance in word reading for children in kindergarten and 1^st^ grade, as compared to 4.1% unique variance for the older 2^nd^-3^rd^ graders. When derivational and compound awareness were both included in these statistical models, only derivational awareness predicted word reading; however, when not competing in the same model, both types of morphological awareness were independently associated with word reading. These findings reveal a robust contribution of morphology to early English word reading at the onset of schooling as well as in slightly more advanced readers. Our results also extend prior work that identified an existing but less consistent association between morphology and early literacy skill (Apel et al., 2013; Law & Ghesquière, 2017).

This result is closely aligned with both the Reading Systems Framework (Perfetti & Stafura, 2014) as well as Psycholinguistic Grain Size Theory (Ziegler & Goswami, 2005). First, the Reading Systems Framework posits that as morphology is integral to each word, it should play an important role in single word identification. Second, Psycholinguistic Grain Size Theory suggests that due to the opacity of English orthography and inconsistent sound-to-print mapping, English readers may need to rely on a larger grain size (e.g., morphemes rather than single phonemes) to identify words. We find strong evidence in support of these theoretical perspectives, revealing that morphological awareness contributes to single word reading, including in kindergarten and 1^st^ grade beginning readers.

There are a number of possible reasons that derivational morphology might be more predictive of English word reading than compound morphology. Children’s understanding of derivational morphology emerges over a more protracted period than compound morphology. Furthermore, derivation is more productive than compounding in English, and derived words become increasingly common in academic texts as children progress through elementary school (Nagy & Anderson, 1984). Knowledge of derived structures may therefore be a more valuable currency for readers as they encounter unfamiliar words. We argue that it is valuable to include compounding in morphology tasks to inform our understanding of morphological awareness in beginning readers; however, when it comes to predicting literacy skill, derivational awareness is more closely related to English word reading.

### 4.3 Morphology and English word reading in Spanish–English and Chinese–English bilinguals

After establishing that morphological awareness contributes to word reading in our sample, we turned to our question about the impact of bilingual experience on the relation between English morphological awareness and English reading. To answer this question, we first identified groups of English monolinguals, Spanish–English bilinguals, and Chinese–English bilinguals with equivalent English vocabulary knowledge and word reading ability. This was because the monolinguals in our sample had higher average English vocabulary knowledge than either bilingual group. Not only are morphology and vocabulary knowledge closely related, but we also recognized the possibility that the role of morphological awareness in word reading may vary, both at different levels of English lexical knowledge, and levels of English literacy. We therefore identified groups that were matched on both their English word reading skill and English vocabulary knowledge in order to hone in specifically on heritage language-specific transfer effects.

#### Performance on derivational versus compound morphology

We predicted that bilingual experiences with derivationally rich Spanish would enhance Spanish–English bilinguals’ performance with English derivations (e.g., extracting *argue* from *argument*), compared to Chinese–English bilinguals (Ramírez et al., 2011). Conversely, we predicted that experience with the compound structure of Chinese would enhance children’s performance with English compounding (e.g., extracting *walk* from *sidewalk*). Interestingly, independent sample *t*-tests revealed no differences between Spanish–English and Chinese–English bilinguals’ accuracy on derived or compound items. This null finding is likely the result of our experimental approach and participant groups. Prior research has revealed differences in compound versus derivational morphology in Spanish- and Chinese-speakers who were learning English (Ramírez et al., 2011); in contrast, our participants had high dual-language proficiency, including age-appropriate English vocabulary and literacy scores. Differences in English morphology may therefore exist between bilingual groups with lower English proficiency levels, but these were not observed in our high proficiency speakers.

#### Contrasting bilingual effects on English word reading

We further predicted that bilingual experience may have a differential effect on how compound and derivational morphological awareness contribute to English reading for Spanish–English and Chinese–English bilingual learners. Specifically, we expected that Spanish–English bilinguals would show a stronger relation between derivational morphology and word reading, while Chinese–English bilinguals would demonstrate a stronger relation between compound morphology and word reading. Indeed, multiple regression revealed significant interactions between morphological item type (derivations vs. compounds) and language group (monolingual vs. Spanish–English bilingual vs. Chinese–English bilingual). This finding supported our overarching hypothesis that experience with structurally distinct languages would alter the relation between morphology and English word reading. Yet the direction of these bilingual effects was contrary to our prediction. Awareness of compound morphology explained significant variance in Spanish–English bilinguals’ word reading, while derivational awareness did not. Conversely, awareness of derivational morphology explained significant variance in Chinese–English bilinguals’ word reading, while compound awareness did not. In other words, differences in English word reading skill among bilingual children was best explained by variation in the type of morphology that was dissimilar or *less* characteristic of a child’s home language.

These findings are consistent with a usage-based hypothesis of language acquisition. The usage-based framework suggests that successful language learning requires that a learner has encountered sufficient examples of a specific linguistic form to be able to make broader generalizations (Ellis, 2002). While a beginning learner may rely heavily on aspects of a new language that can be transferred from their L1, a more advanced learner requires explicit instruction in the unique aspects of the second language that cannot be transferred. It is experiences with less frequent structures – for instance, the structures that are unique to a single language and not shared across a bilingual’s two languages – that are necessary to drive additional growth. For the highly proficient bilinguals in our present study, variance in English reading depends on children’s proficiency with the features of English that cannot be gleaned from their home language.

#### Heritage language morphology and English word reading

Finally, we examined the role of children’s morphological awareness in their heritage language in their English word reading. Even when accounting for Spanish or Chinese morphological awareness, we continued to observe a close association between English compound awareness and word reading for Spanish bilinguals, and a contrasting association between English derivational awareness and word reading for Chinese bilinguals. These results extend and clarify the discovery that bilinguals’ English word reading is associated with their sensitivity to the morphological structures that are *less characteristic* of their home language. Additionally, we find that morphological awareness measured in Spanish contributes to Spanish bilinguals’ English word reading, whereas morphological awareness measured in Chinese does not. This result is perhaps related to the linguistic distance between English and Chinese, which makes it more difficult to transfer Chinese morphological awareness directly to support English literacy.

These findings are also aligned with recent neurocognitive evidence suggesting language-specific bilingual differences in morphological processing. Sun and colleagues (2022) examined the brain bases of English morphology in Spanish–English and Chinese–English bilinguals with high English literacy, a sample that partially overlapped with the current study. Participants completed an auditory oddball task in which they heard three English words, and indicated which two words shared a morpheme. This task included compound words with shared root morphemes (e.g., *spaceship, battleship, friendship*) as well as items with shared derivational affixes (e.g., *disagree,* *dishonest, distance*). fNIRS neuroimaging revealed that bilinguals with greater Spanish or Chinese vocabulary knowledge showed increased left superior temporal brain activation when processing the English words that were less characteristic of their home language (i.e., derivations for Chinese bilinguals). Notably, home language proficiency was not associated with brain activation during derivational processing for Spanish bilinguals, nor during compound processing for Chinese bilinguals. Together with the present study, these converging neuro-behavioral observations speak to the contrasting, language-specific effects of bilingual proficiency on sub-lexical processes that support learning to read.

In sum, our findings reveal linguistically principled bilingual effects on word reading in high proficiency bilinguals, and suggest that greater familiarity with linguistic features that are dissimilar across the children’s two languages may bolster reading success. These findings both support and extend theories of bilingual language transfer (Chung et al., 2019). For bilingual children who are still acquiring their language of schooling (e.g., Ramírez et al., 2011), their established proficiency in L1 likely contributes to and scaffolds their emerging proficiency in L2. For bilinguals with advanced levels of dual language proficiency, additional gains are likely driven by greater attention to those elements of language that are maximally dissimilar across languages. The observed bilingual differences are thus consistent with the idea that bilinguals’ two languages interact to influence literacy (Interdependence Continuum; Proctor et al., 2010), and will manifest continuously throughout development but differently at varying stages of reading proficiency, both as a function of dual language proficiency and cross-linguistic experiences (Interactive Transfer Framework; Chung et al., 2019).

## 5. Broader implications and future directions

The present study demonstrates that linguistically diverse children may follow different paths to literacy success. In particular, bilingual children’s path to successful literacy may vary as a function of their specific home language. In support of this idea, we found different associations between children’s English language and literacy skills across our three language groups. For instance, phonological awareness and word reading were more strongly correlated among Spanish–English bilinguals, while vocabulary and word reading were more strongly correlated among Chinese–English bilinguals. These findings extend prior work demonstrating greater reliance on sound-based processes for Spanish bilinguals (Kremin, Arredondo, Hsu, Satterfield & Kovelman, 2016), as compared to greater reliance on meaning-based processes for Chinese bilinguals (Hsu, Ip, Arredondo, Tardif & Kovelman, 2016). Our findings also beg the question: what other heritage language competencies are bilingual children bringing into the classroom to support their reading development? This is a critical area for continued study, as a growing body of research demonstrates that bilingual children’s home language knowledge is a resource that benefits learning (Durgunoğlu, Segers & Broek, 2017).

At an applied level, our findings add to the growing body of literature demonstrating the importance of morphological awareness for learning to read, and reinforce the idea that explicit morphological instruction may benefit young learners. Indeed, short morphological interventions have been linked with improved long-term reading outcomes (Lyster, Lervåg & Hulme, 2016), and a meta-analysis suggests that morphological instruction may have the greatest impact for young learners (Goodwin & Ahn, 2013). The present study provides some preliminary insight into how this morphological instruction might be individualized across diverse learners. Our findings may help inform instruction by suggesting that proficient or balanced bilingual children may benefit from learning activities that bolster their sensitivity to less familiar English structures.

This manuscript has several caveats. Our study lacks an explicit measure of multimorphemic word reading. Although a major advantage of the current reading measures is their standardization, future research may benefit from careful consideration of multimorphemic word or pseudoword reading tasks (e.g., Hasenäcker et al., 2017). Additionally, as our data capture a single point in time, we are unable to draw conclusions about causal mechanisms.

Although our sample is ethnically, linguistically and geographically diverse, participants come from families of predominantly middle-to-high socioeconomic and education status. On one hand, this is a particularly important limitation given that bilingual learners in the United States often grow up in homes with a lower socioeconomic status, which has a well-documented impact on language development (Hoff et al., 2012). Both monolingual and bilingual participants in our sample have above-average English vocabulary knowledge, which may limit generalizability. On the other hand, this unique sample may serve to dissociate bilingual experiences from the confound of SES, providing insight into cross-linguistic influences on literacy. Other possible unaddressed confounds include dialectal differences in heritage language, as well as schooling context: although some participants in our sample are enrolled in dual language immersion programs, we are unable to tease apart how bilingual instruction may influence morphological knowledge. Additional studies are needed with more diverse samples, and across socio-economic, socio-cultural, and academic contexts to broaden our understanding of how bilingualism impacts literacy.

This manuscript offers both theoretical and practical implications for understanding the role of morphology in reading development. First, our findings extend current theoretical perspectives on English reading acquisition by demonstrating that children’s morphological awareness contributes to their literacy achievement as early as age 5. Furthermore, we reveal principled bilingual transfer effects on the association between children’s morphological awareness and their word reading skill, advancing our understanding of bilingual literacy among children with early and systematic dual language exposure. Remarkably, our findings reveal mechanistic differences in the contribution of morphology to word reading, even among a sample of Spanish–English and Chinese–English bilingual children with equivalent and highly proficient English skill. These findings reinforce the notion that, even in the case of dual-first language learners with high proficiency in the language of schooling, the bilingual mind cannot be understood as the sum of two monolinguals (Grosjean, 1989).

## Supplementary Material

Supplement ELMM Task

Supplementary Tables

## Figures and Tables

**Table 1.** Descriptive Statistics and Correlations Between Language and Literacy Measures

	*M*	*SD*	Range	*1*	*2*	*3*	*4*	*5*	*6*	*7*
1. Age	7.39	1.06	5–9	-						
2. Vocabulary^a^	112.75	15.66	85–160	.64	-					
3. Phonological awareness^b^	11.26	2.71	3–20	.43	.42	-				
4. Single word reading^a^	110.58	16.49	66–145	.61	.52	.70	-			
5. Reading comprehension^a^	105.23	13.55	64–140	.61	.50	.65	.87	-		
6. Total ELMM (*N* = 40)	24.79	9.40	0–39	.65	.66	.59	.69	.71	-	
7. Early-acquired derivations	9.08	3.51	0–13	.61	.60	.66	.63	.66	.93	-
8. Early-acquired compounds	9.37	3.10	0–13	.56	.51	.50	.59	.60	.89	.76

Note.

a Standard score with population mean of 100, *SD* = 15.

b Scaled score with population mean of 10 (typical range: 8–12).

Correlations were conducted with raw scores. All correlations are significant at the *p* < 0.001 level (2-tailed).

**Table 2.** Hierarchical Regression Predicting Single Word Reading

	*p*	*t*	*p*	*R*	*R* ^2^	Δ *R*^2^
Step 1				.617	.375	
Constant		−4.65	<.001			
Age	.62	13.90	<.001			
Bilingual status	.15	3.16	.002			
Maternal education	.14	3.25	.001			
Step 2				.798	.636	.255
Constant		−6.04	<.001			
Age	.30	6.51	<.001			
Bilingual status	.16	4.46	<.001			
Maternal education	.05	1.41	.160			
Vocabulary	.16	3.41	<.001			
Phonological awareness	.51	13.41	<.001			
Step 3				.821	.675	.039
Constant		−3.36	.001			
Age	.19	4.17	<.001			
Bilingual status	.19	5.56	<.001			
Maternal education	.04	1.18	.241			
Vocabulary	.07	1.45	.147			
Phonological awareness	.41	10.29	<.001			
Derivational MA	.23	3.61	<.001			
Compound MA	.11	2.01	.045			

*Note.* MA=morphological awareness. Final model explains significant unique variance in single word reading, *F*(7, 321) = 95.15, *p* < .001.

**Table 3.** Descriptive Statistics and ANOVA Testing for Differences in English Language and Literacy Raw Scores Across Language Groups

	English monolinguals		Spanish-English bilinguals		Chinese-English bilinguals		*F*	*p*
	*M*	*SD*	*M*	*SD*	*M*	*SD*		
Age	7.38	1.05	7.64	1.03	7.04	1.19	5.27	.006**
Maternal education^a^	9.04	1.67	8.21	2.26	9.39	2.03	6.02	.003**
English vocabulary	147.16	17.64	142.30	21.33	139.74	25.29	2.19	.114
Phonological awareness	22.88	6.51	22.71	7.72	22.32	6.86	0.11	.893
Derivational morphology	9.72	3.21	8.35	3.65	8.51	3.77	3.10	.047*
Compound morphology	9.78	2.90	9.25	2.98	8.81	3.40	1.70	.185
Word reading	46.51	13.56	47.43	14.11	47.52	13.85	0.11	.892
Reading comprehension	25.79	7.61	24.08	7.25	25.67	7.21	1.13	.326
Spanish/Chinese vocabulary standard score	-	-	108.60	16.20	95.90	17.39	2.89	.091

Note.

a Educational attainment scale: 8 = Completed bachelor’s degree; 9 = Some graduate school; 10 = Completed master’s degree. English language and literacy skills are all presented as raw scores for comparison across groups of slightly different ages.

**Table 4.** Intercorrelations Between Literacy Variables by Language Group

	1	2	3	4	5	6
English monolinguals (*N* = 69)						
1. English vocabulary	-					
2. Phonological awareness	.50***	-				
3. Single word reading	.74***	.62***	-			
4. Reading comprehension	.71***	.71***	.91***	-		
5. Derivational morphology	.63***	.67***	.79***	.79***	-	
6. Compound morphology	.59***	.52***	.66***	.65***	.71***	-
Spanish-English bilinguals (*N* = 69)						
1. English vocabulary	-					
2. Phonological awareness	.47***	-				
3. Single word reading	.58***	.80***	-			
4. Reading comprehension	.72***	.67***	.85***	-		
5. Derivational morphology	.64***	.55***	.61***	.67***	-	
6. Compound morphology	.57***	.63***	.70***	.68***	.74***	-
7. Spanish vocabulary	.59***	.28**	.46***	.53***	.35**	.41**
Chinese-English bilinguals (*N* = 69)						
1. English vocabulary	-					
2. Phonological awareness	.51***	-				
3. Single word reading	.74***	.73***	-			
4. Reading comprehension	.72***	.63***	.86***	-		
5. Derivational morphology	.74***	.58***	.81***	.79***	-	
6. Compound morphology	.53***	.45***	.60***	.58***	.74***	-
7. Chinese vocabulary	.23^†^	.18	.14	.22^†^	.16	.26*

Note.

† *p* < .10

* *p* < .05

** *p* < .01

*** *p* < .001.

**Table 5.** Regression Predicting Word Reading from Morphology × Language Group Interaction

Reference group: Spanish bilinguals					Reference group: Chinese bilinguals				
	*β*	*t*	*p*			*β*	*t*	*p*	
Constant	−1.75	−3.85	<.001	***	Constant	−1.62	−3.76	<.001	***
Age	0.24	4.05	<.001	***	Age	0.24	4.05	<.001	***
Maternal education	0.13	2.87	.005	**	Maternal education	0.13	2.87	.005	**
Vocabulary	0.19	2.86	.005	**	Vocabulary	0.19	2.86	.005	**
LG: Chinese	0.13	1.22	.224		LG: Spanish	−0.24	−1.22	.224	
LG: English	−0.25	−2.57	.019	*	LG: English	−0.38	−3.77	<.001	***
Derivations	−0.01	−0.10	.918		Derivations	0.37	3.24	.001	***
Compounds	0.48	4.14	<.001	***	Compounds	0.07	0.71	.481	
Chinese × Derivations	0.39	2.55	.011	*	Spanish × Derivations	−0.39	−2.55	.011	*
English × Derivations	0.55	3.53	.001	***	English × Derivations	0.17	1.08	.282	
Chinese × Compounds	−0.42	−2.70	.008	**	Spanish × Compounds	0.42	2.70	.008	**
English × Compounds	−0.39	−2.47	.014	*	English × Compounds	0.03	0.20	.846	

*Note.* LG = Language Group. *F*(11, 191)=38.27, *p* < .001, adjusted *R^2^* = 0.67.

**Table 6.** Regression Analysis Predicting English Word Reading in Spanish Bilinguals

	B	β	T	p
(Constant)	5.77		1.08	.285
English derivations	0.97	.23	1.67	.102
English compounds	1.64	.35	2.38	.021 *
Spanish morphological awareness	0.31	.34	3.17	.003 **

*Note.* Model explains significant unique variance, *F*(3, 48) = 25.03, *p* < .001.

**Table 7.** Regression Analysis Predicting English Word Reading in Chinese Bilinguals

	B	β	T	p
(Constant)	17.19		2.82	.008
English derivations	3.03	.77	4.89	<.001 ***
English compounds	−0.15	−.04	−0.23	.818
Chinese morphological awareness	0.12	.12	1.12	.268

*Note.* Model explains significant unique variance, *F*(3, 39) = 22.27, *p* < .001.
