# Supplementary material for: Morphological awareness and its role in early word reading in English monolinguals, Spanish–English, and Chinese–English simultaneous bilinguals
Source: Biling (Camb Engl). Author manuscript; Available in PMC 2024 Mar 1. (PMC10103835; doi:10.1017/s1366728922000517)
Supplement: Supplement ELMM Task [file NIHMS1842613-supplement-Supplementary_Tables.pdf]

Appendix

Early Lexical Morphology Measure (ELMM)

Administration: Read instructions and 40 test items to the child. If child provides incorrect or no response, record verbatim. Also note self-corrections.
Feedback: Provide feedback only on training. Tester can repeat each item once if requested.
Ceiling: 10 incorrect in a row.
Suggested prompts: How should we finish that sentence? Do you want to take your best guess?

Instructions: We're going to play a game with sentences. I will say a word, and then you are going to use part of that word to help me finish a sentence. Ready?

Training:

A. Friendly She is my best ____________________. Friend E

If correct: Right! We can take part of the word "friendly," and turn it into the word "friend" to finish that sentence. Let's try another one. **Proceed to Item 1.**

If incorrect: Remember, we're going to use the word I say to finish the sentence. If I say "friendly," we can use part of that word to make "friend." She is my best *friend*. Let's try another. **Go to training item B.**

B. Playful Let's go outside and ____________________. Play E

If correct: Right! We can take part of the word "playful," and turn it into the word "play" to finish that sentence. Let's try another one. **Proceed to Item 1.**

If incorrect: Remember, we're going to use the word I say to finish the sentence. If I say "playful," we can use part of that word to make "play." Let's go outside and *play*. Let's try another one. **Proceed to Item 1.**

Test items 1-40. No feedback.

1.	**Foggy**	On some mornings, you can see _______________.	Fog	E
2.	**Runner**	My sister and I went on a _______________.	Run	E
3.	**Teamwork**	This weekend, my dad has to _______________.	Work	E
4.	**Football**	Ouch! You stepped on my _______________.	Foot	E
5.	**Quickly**	That lion was _______________.	Quick	E
6.	**Teaspoon**	I eat my soup with a _______________.	Spoon	E
7.	**Sidewalk**	The baby is learning how to _______________.	Walk	E
8.	**Noisy**	Did you hear that _______________?	Noise	E
9.	**Colorful**	That flower is such a pretty ____________________.	Color	E

10. **Classroom**	Go upstairs and clean your ____________________.	Room	E
11. **Stroller**	Would you like to go for a ____________________?	Stroll	E
12. **Sensitive**	He wasn't making any ____________________.	Sense	E
13. **Awesome**	She looked at the ocean with ____________________.	Awe	E
14. **Computer**	The distance from here to Jupiter is hard to ___________.	Compute	E
15. **Personality**	George Washington was a famous _______________.	Person	E
16. **Careful**	Those glasses look breakable! Handle them with __________.	Care	E

You are doing a great job! Let's keep going. Remember, we're going to use part of the word I say to finish the sentence.

17. **Blueberry**	My favorite shirt is _______________.	Blue	E
18. **Election**	How many women did they _______________?	Elect	E
19. **Backyard**	I forgot my jacket, so I have to go _______________.	Back	E
20. **Argument**	My coach told me not to _______________.	Argue	E
21. **Rainbow**	I like to go outside in the _______________.	Rain	E
22. **Breakfast**	It's time to take a _______________.	Break	E
23. **Correction**	How many mistakes did the teacher _______________?	Correct	E
24. **Raincoat**	Before I play in the snow, I put on my _______________.	Coat	E
25. **Curiosity**	Cats are always _______________.	Curious	E
26. **Breathe**	In the winter, sometimes you can see your _____________.	Breath	E
27. **Height**	That box is too _______________.	High	E
28. **Seaweed**	The mermaid lives in the _______________.	Sea	E
29. **Necklace**	Let's tie it together with _______________.	Lace	E
30. **Discussion**	What did she want to _______________?	Discuss	E
31. **Vacation**	The police told us we have to _______________.	Vacate	E
32. **Warmth**	At night, my room gets too _______________.	Warm	E
33. **Combination**	Which colors should the painter _______________?	Combine.	E
34. **Afternoon**	I'm reading right now, but we can play _____________.	After	E

35.	**Elasticity**	That rubber band feels ________________.	Elastic	E
36.	**Length**	The river is very ________________.	Long	E
37.	**Somebody**	My dog has big ears and a little ________________.	Body	E
38.	**Decision**	Which game should we play? I can't ________________.	Decide	E
39.	**Strengthen**	Fire fighters have to be really ________________.	Strong	E
40.	**Remarkable**	Did you hear his ________________?	Remark	E

Total # incorrect: ________

Raw score: _________ / 40

Compounds	Derivations	
1. Rainbow / rain*	1. Foggy / fog††	16. Height / high*†† (P)
2. Football / foot*	2. Runner / run*†	17. Discussion / discuss†† (P)
3. Teaspoon / spoon*	3. Quickly / quick*	18. Vacation / vacate (P)
4. Sidewalk / walk*	4. Noisy / noise*	19. Warmth / warm*†
5. Classroom / room*	5. Colorful / color*	20. Combination / combine† (P)
6. Seaweed / sea*	6. Stroller / stroll	21. Elasticity / elastic (P)
7. Blueberry / blue*	7. Sensitive / sense†	22. Length / long* (P)
8. Backyard / back*	8. Computer / compute	23. Decision / decide (P)
9. Teamwork / work*	9. Personality / person*	24. Strength / strong* (P)
10. Breakfast / break* (P)	10. Careful / care*†	25. Remarkable / remark††
11. Raincoat / coat*	11. Election / elect†† (P)	
12. Somebody / body* (P)	12. Argument / argue*	
13. Afternoon / after*	13. Correction / correct* (P)	
14. Necklace / lace (P)	14. Curiosity / curious (P)	
15. Awesome / awe	15. Breathe / breath*† (P)	

Note. *Early acquired root word. (P) = phonological shift required to correctly produce root word.
†Item modified from Goodwin et al.'s (2012) Extract the Base task. ††Verbatim item in Goodwin et al.'s (2012) Extract the Base task.
