# Supplementary material for: Morphological awareness and its role in early word reading in English monolinguals, Spanish–English, and Chinese–English simultaneous bilinguals
Source: Biling (Camb Engl). Author manuscript; Available in PMC 2024 Mar 1. (PMC10103835; doi:10.1017/s1366728922000517)
Supplement: Supplementary Tables [file NIHMS1842613-supplement-Supplement_ELMM_Task.pdf]

Morphological awareness and its role in early word reading in English monolinguals, Spanish-English, and Chinese-English simultaneous bilinguals: Supplemental Materials

Marks, Labotka, Sun, Nickerson, Zhang, Eggleston, Yu, Uchikoshi, Hoeft & Kovelman

Contents of Supplement:

Table S1. *Demographic Characteristics of Participants in Study 1 and Study 2* 2

Study 1: Associations Between Morphology and Word Reading

Table S2. *Study 1 Participants' Language Background by Grade and by Site* 3

Table S3. *Mean Accuracy on ELMM Items by Grade and Language Background* 3

Table S4. *Hierarchical Regression Predicting Word Reading in K-1st Graders* 4

Table S5. *Hierarchical Regressions Predicting Word Reading in K-1st Graders from Derivational Awareness and Compound Awareness Separately* 4

Table S6. *Hierarchical Regression Predicting Word Reading in 2nd-3rd Graders* 5

Table S7. *Hierarchical Regressions Predicting Word Reading in K-1st Graders from Derivational Awareness and Compound Awareness Separately* 5

Study 2: Bilingual Transfer Effects on Morphological Awareness and English Reading

The role of heritage language morphological awareness in English word reading 6

Table S8. *Regression analysis predicting English word reading in Spanish bilinguals* 7

Table S9. *Regression analysis predicting English word reading in Chinese bilinguals* 7

Supplemental Table 1
Demographic Characteristics of Participants in Study 1 and Study 2

	Study 1 (*n* = 340)		Study 2 (*n* = 207)	
Variable	n	%	n	%
Sex				
Male	190	54.3	110	53.1
Female	160	45.7	97	46.9
Grade				
Pre-K – K	70	20.0	50	24.2
1st	137	39.1	77	37.2
2nd	93	26.6	50	24.2
3rd	50	14.3	30	14.5
Geographic location				
Site 1	238	68.0	157	75.8
Site 2	112	32.0	50	24.2
Language				
English monolingual	175	50.0	69	33.3
Spanish-English bilingual	78	22.3	69	33.3
Chinese-English bilingual	89	25.4	69	33.3
Other home language(s)	9	2.6	0	0
Race and ethnicity				
Asian	96	27.4	63	30.4
Black or African American	7	2.0	5	2.4
Hispanic or Latinx	71	20.3	58	28.0
Multiracial or Multiethnic	55	15.7	35	16.9
White or European American	121	34.6	46	22.2
Maternal educational attainment				
No high school diploma	6	1.7	6	2.9
High school or GED	10	2.9	6	2.9
Some college	14	4.0	8	3.8
Associate's degree	11	3.1	5	2.4
Bachelor's degree	106	30.3	64	31.5
Some graduate school	9	2.6	6	2.9
Master's degree	139	39.7	76	36.7
Professional or doctoral degree	51	14.6	32	15.5
Missing data	4	1.1	4	1.9

Study 1: Associations Between Morphology and Word Reading

Supplemental Table 2

Study 1 Participants' Language Background by Grade and by Site

	English monolinguals		Spanish-English bilinguals		Chinese-English bilinguals		Other home language		Total
Grade	*N*	%	*N*	%	*N*	%	*N*	%	*N*
Pre-K – K	27	38.6	16	22.9	27	38.6	0	-	70
1st grade	60	43.8	34	24.8	38	27.7	5	3.6	137
2nd grade	35	37.6	31	33.3	23	24.7	4	4.3	93
3rd grade	21	42.0	16	32.0	13	26.0	0	-	50
Site									
Site 1	100	42.0	66	27.7	72	30.3	0	-	238
Site 2	43	38.4	31	27.7	29	25.9	9	8.0	112

Supplemental Table 3

Mean Accuracy on ELMM Items by Grade and Language Background

	**Early Acquired Derivations**				**Early Acquired Compounds**			
	English monolinguals	Spanish bilinguals	Chinese bilinguals	All	English monolinguals	Spanish bilinguals	Chinese bilinguals	All
Pre-K – K	5.41 (3.52)	4.13 (2.39)	4.28 (2.56)	4.84 (3.06)	6.59 (3.19)	6.00 (2.16)	6.00 (2.48)	6.34 (2.73)
1st grade	9.50 (2.74)	8.16 (3.26)	8.93 (3.34)	9.25 (2.95)	9.33 (2.74)	8.52 (2.32)	8.83 (3.76)	9.15 (3.00)
2nd grade	10.97 (2.07)	8.80 (3.83)	9.94 (2.84)	10.36 (2.78)	10.73 (2.04)	10.40 (2.21)	10.78 (3.02)	10.77 (2.23)
3rd grade	11.90 (1.22)	10.31 (3.13)	12.38 (0.87)	11.52 (2.13)	11.86 (1.49)	10.75 (2.38)	11.08 (1.66)	11.30 (1.89)
	**All Derivations**				**All Compounds**			
Pre-K – K	7.89 (5.12)	6.81 (4.02)	5.88 (4.22)	7.14 (4.77)	6.59 (3.19)	6.00 (2.16)	6.04 (2.52)	6.39 (2.79)
1st grade	15.93 (4.82)	13.36 (5.67)	14.23 (5.78)	15.20 (5.23)	9.58 (2.99)	8.56 (3.28)	9.20 (4.06)	9.38 (3.21)
2nd grade	18.71 (3.98)	14.85 (7.01)	16.17 (5.68)	17.47 (5.23)	11.41 (2.45)	10.55 (2.35)	11.28 (3.39)	11.30 (2.60)
3rd grade	20.57 (2.64)	18.50 (4.53)	21.85 (1.82)	20.24 (3.41)	12.76 (2.10)	11.06 (2.62)	12.15 (2.27)	12.06 (2.39)

Supplemental Table 4

Hierarchical Regression Predicting Word Reading in K-1st Graders

	β	*t*	*p*	*R*	R^2	ΔR^2
Step 1				.522	.272	
Constant		-3.52	.001			
Age	.51	8.04	<.001			
Bilingual status	.22	3.51	.001			
Maternal education	.06	0.91	.363			
Step 2				.733	.537	.264
Constant		-4.36	<.001			
Age	.28	4.81	<.001			
Bilingual status	.24	4.44	<.001			
Maternal education	.04	0.72	.470			
Vocabulary	.11	1.80	.073			
Phonological awareness	.52	9.54	<.001			
Step 3				.763	.582	.045
Constant		-2.33	.021			
Age	.17	2.84	.005			
Bilingual status	.28	5.23	<.001			
Maternal education	.03	0.59	.556			
Vocabulary	.05	0.77	.445			
Phonological awareness	.41	7.06	<.001			
Derivations	.24	2.72	.007			
Compounds	.09	1.14	.255			

Note. Final model explains significant variance in word reading, $F(7,185) = 36.76$, $p < .001$

Supplemental Table 5

Hierarchical Regressions Predicting Word Reading in K-1st Graders from Derivational Awareness and Compound Awareness Separately

	β	*t*	*p*	β	*t*	*p*
Constant		-2.23	.027		-3.65	<.001
Age	.18	2.88	.004	.22	3.74	<.001
Bilingual status	.28	5.29	<.001	.26	4.77	<.001
Maternal education	.03	0.51	.608	.04	0.81	.419
Vocabulary	.05	0.77	.444	.08	1.29	.198
Phonological awareness	.41	7.14	<.001	.24	8.13	<.001
Derivations	.30	4.31	<.001			
Compounds				.21	3.49	<.001

Supplemental Table 6

Hierarchical Regression Predicting Word Reading in 2nd-3rd Graders

	β	*t*	*p*	*R*	R^2	ΔR^2
Step 1				.422	.178	
Constant		-0.05	.957			
Age	.26	3.33	.001			
Bilingual status	.08	1.01	.316			
Maternal education	.34	4.21	<.001			
Step 2				.776	.603	.425
Constant		-0.94	.351			
Age	.07	1.24	.217			
Bilingual status	.09	1.49	.140			
Maternal education	.08	1.33	.186			
Vocabulary	.25	3.86	<.001			
Phonological awareness	.60	9.75	<.001			
Step 3				.802	.644	.041
Constant		-0.26	.797			
Age	.04	0.72	.476			
Bilingual status	.11	2.04	.043			
Maternal education	.06	0.98	.327			
Vocabulary	.13	1.73	.085			
Phonological awareness	.51	8.20	<.001			
Derivations	.21	2.33	.021			
Compounds	.10	1.46	.147			

Note. Final model explains significant variance in word reading, $F(7,135) = 33.05$, $p < .001$.

Supplemental Table 7

Hierarchical Regressions Predicting Word Reading in 2nd-3rd Graders from Derivational Awareness and Compound Awareness Separately

	β	*t*	*p*	β	*t*	*p*
Constant		0.11	.910		-1.20	.233
Age	.04	0.63	.533	.07	1.15	.251
Bilingual status	.11	1.98	.050	.10	1.82	.072
Maternal education	.06	1.03	.306	.07	1.13	.261
Vocabulary	.12	1.57	.118	.22	3.32	.001
Phonological awareness	.53	8.50	<.001	.54	8.66	<.001
Derivations	.28	3.54	<.001	-	-	-
Compounds	-	-	-	.19	3.01	.003

Study 2: Bilingual Transfer Effects on Morphological Awareness and English Reading

The role of heritage language morphological awareness in English word reading

As part of a larger project, some participants in the current study completed an additional morphological awareness task in Spanish or Chinese. This was an oddball task, in which children heard three words: two that shared a morpheme (e.g. class<u>room</u> and bed<u>room</u>), and one with a phonological distractor (e.g., mush<u>room</u>). In Spanish, an example triplet includes the words *automóvil* (automobile), *autopartes* (car parts), and the distractor *autoridad* (authority). In Chinese, an example triplet includes the words 眼镜 (*yan3 jing4*, eyeglasses), 墨镜 (*mo4 jing4*, sunglasses), and the distractor 安静 (*an1 jing4*, quiet). These example items all demonstrate triplets in which two words share a root morpheme; however, the tasks also contain items that share a derived affix.

Of the bilingual children in the matched groups of Study 2, N = 52 Spanish bilinguals (M accuracy = 60.04%, SD = 16.62) and N = 43 Chinese bilinguals (M accuracy = 60.10%, SD = 14.69) completed this oddball task in their heritage language. There were no significant differences in accuracy between groups, $t(93) = -0.02$, $p = .984$.

These data are unfortunately not available for all bilinguals in the current sample. Furthermore, because these tasks are designed in two different languages and completed by two different samples of participants, we cannot guarantee that the heritage language tasks are perfectly matched in the difficulty of each item. However, the incomplete data available suggest that the bilingual groups are well-matched in terms of their heritage language proficiency.

We then examined how heritage language morphological awareness might contribute to English word reading skill, after taking into account the contribution of English morphological awareness. We conducted two separate regression analyses, one for the Spanish-English bilinguals and one for the Chinese-English bilinguals who had completed the heritage language morphology task.

Among Spanish-English bilinguals, English compound awareness and Spanish morphological awareness, but not English derivational awareness, were significantly associated with English word reading (Supplemental Table 1). Among Chinese-English bilinguals, only English derivational awareness was significantly associated with English word reading (Supplemental Table 2).

These results extend and clarify the findings of Study 2, which reveal that participants' sensitivity to the type of English morphology that is *less characteristic* is associated with differences in their English word reading. In this supplemental analysis, we see that the interaction pattern discovered in Study 2 holds even when heritage language proficiency is included in the model. Additionally, we find that morphological awareness measured in Spanish contributes to Spanish bilinguals' English word reading, whereas morphological awareness measured in Chinese does not. This result is perhaps related to the linguistic distance between English and Chinese, which makes it more difficult to transfer Chinese morphological awareness directly to support English literacy.

Supplemental Table 8

Regression analysis predicting English word reading in Spanish bilinguals.

	B	*β*	*t*	*p*
(Constant)	5.77		1.08	.285
English derivations	0.97	.23	1.67	.102
English compounds	1.64	.35	2.38	.021 *
Spanish morphological awareness	0.31	.34	3.17	.003 **

Note. Model explains significant unique variance, $F(3, 48) = 25.03$, $p < .001$.

Supplemental Table 9

Regression analysis predicting English word reading in Chinese bilinguals.

	B	*β*	*t*	*p*
(Constant)	17.19		2.82	.008
English derivations	3.03	.77	4.89	<.001 ***
English compounds	-0.15	-.04	-0.23	.818
Chinese morphological awareness	.12	.12	1.12	.268

Note. Model explains significant unique variance, $F(3, 39) = 22.27$, $p < .001$.
